# Supplementary figures and images for: CRISPR-Cas-Guided Mutagenesis of Chromosome and Virulence Plasmid in Shigella flexneri by Cytosine Base Editing
Source: mSystems. 2022 Dec 21;8(1):e01045-22. doi: 10.1128/msystems.01045-22 (PMC9948704; doi:10.1128/msystems.01045-22)

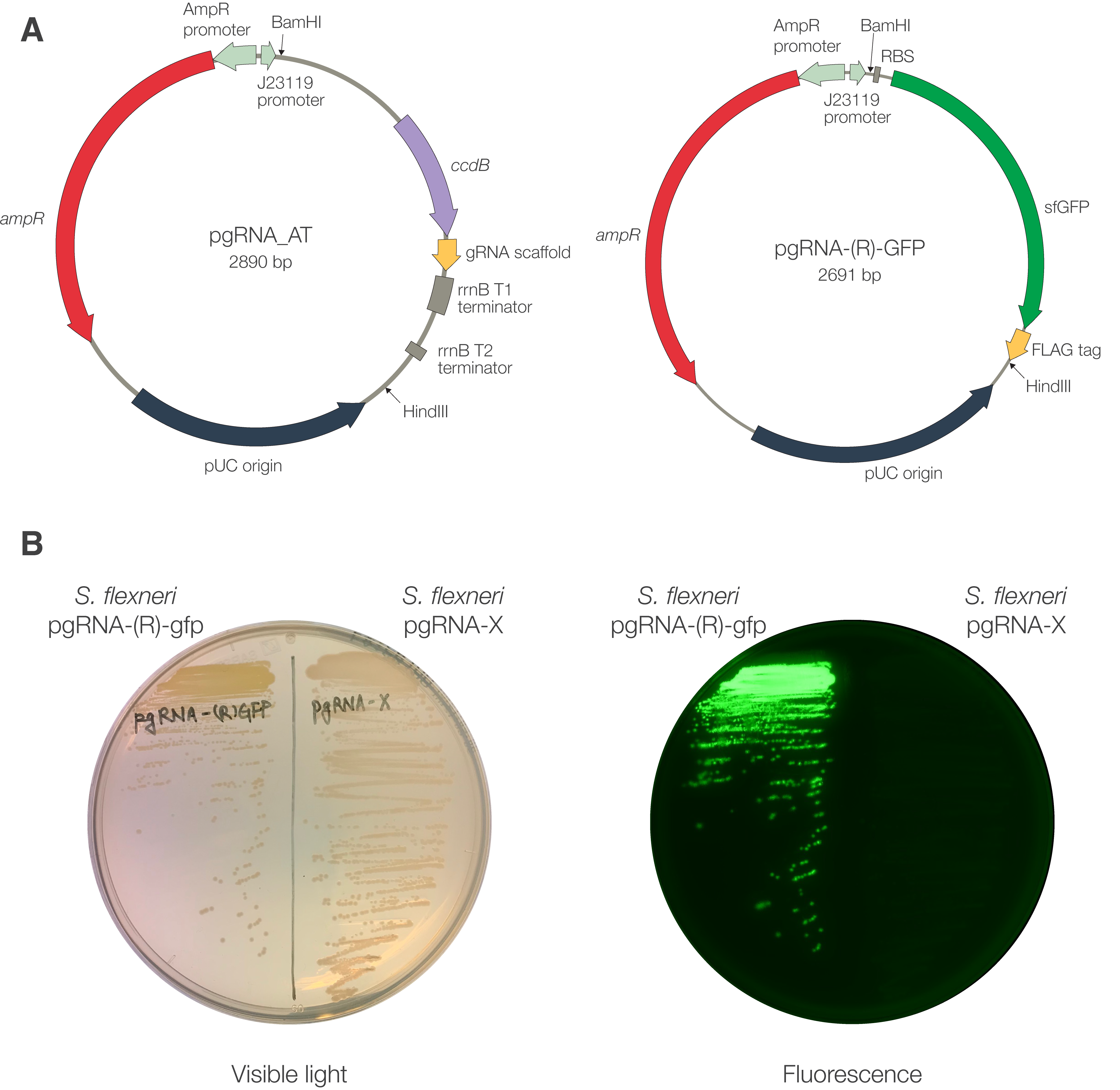

Supplement: FIG S1 [file msystems.01045-22-sf001.tif]

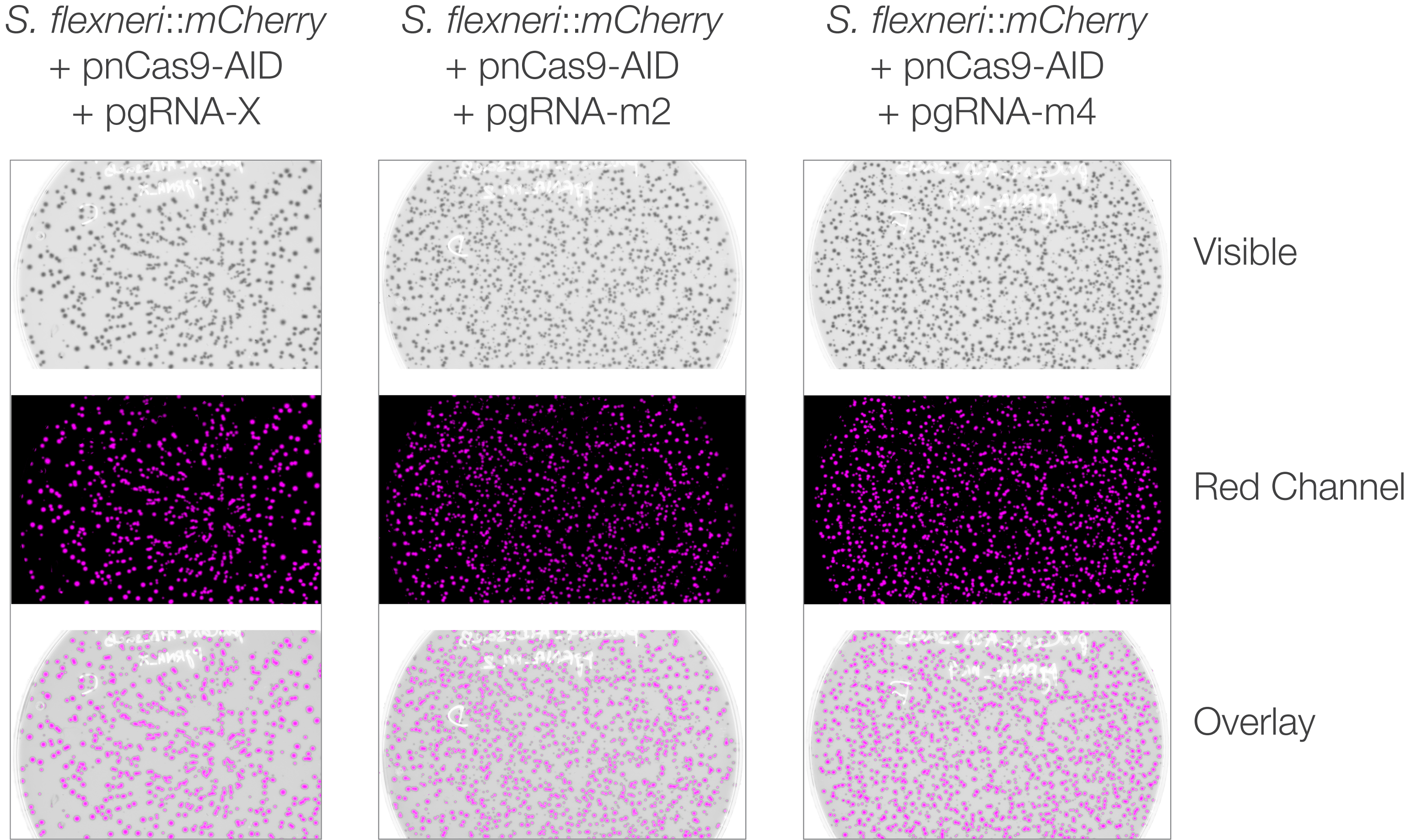

Supplement: FIG S2 [file msystems.01045-22-sf002.tif]

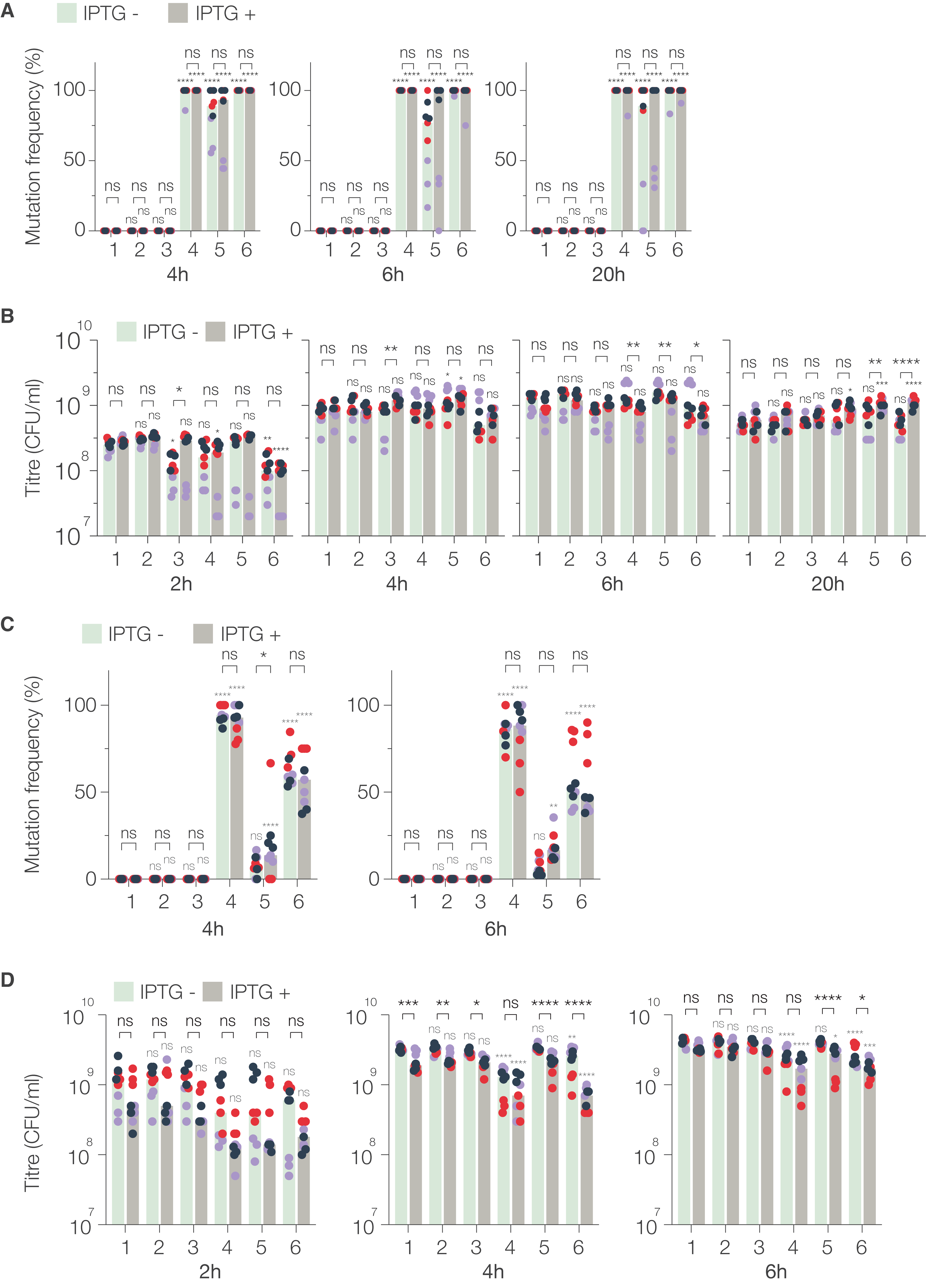

Supplement: FIG S3 [file msystems.01045-22-sf003.tif]

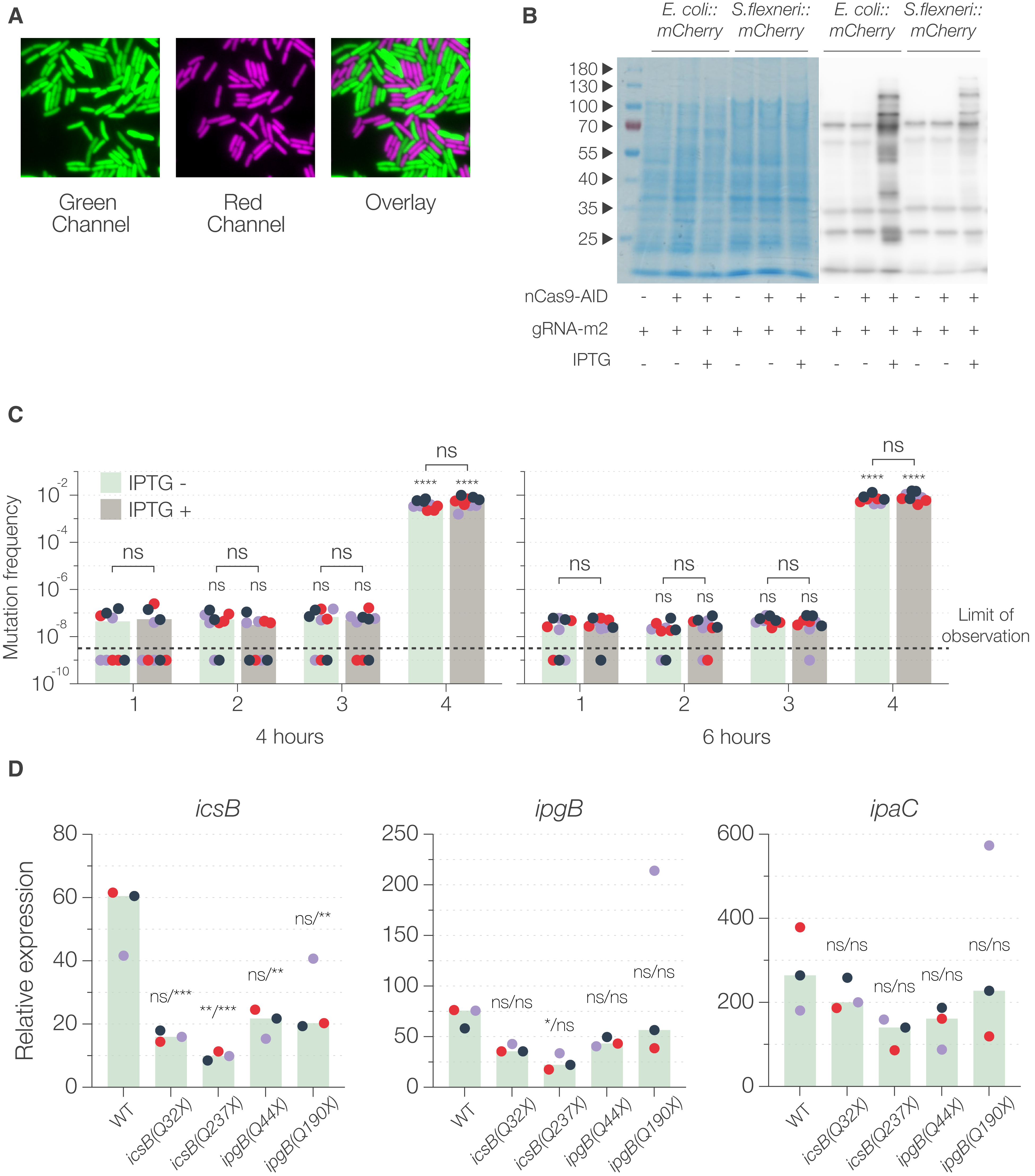

Supplement: FIG S4 [file msystems.01045-22-sf004.tif]

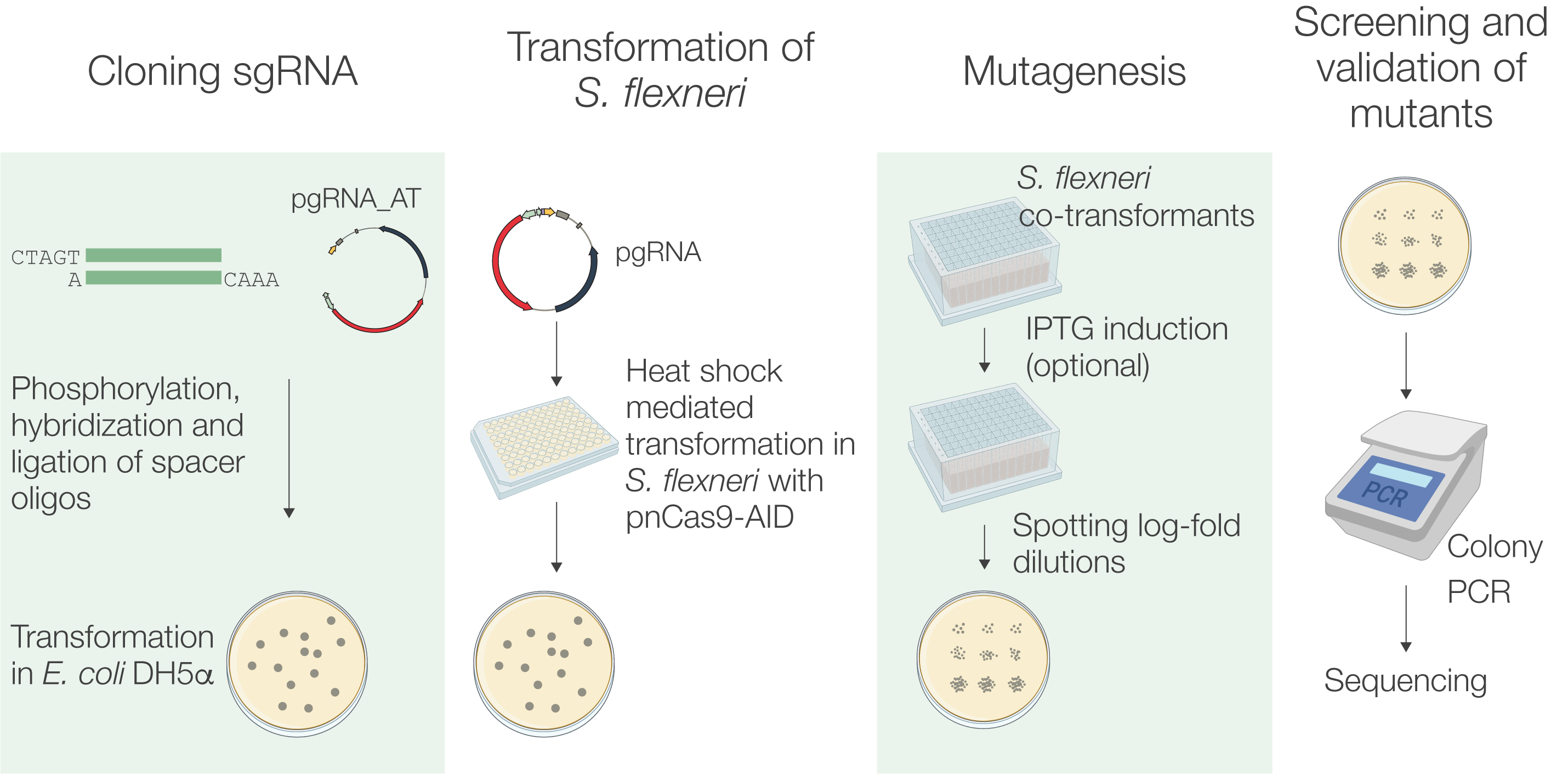

Supplement: FIG S5 [file msystems.01045-22-sf005.tif]
