# Supplementary material for: CRISPR-Cas-Guided Mutagenesis of Chromosome and Virulence Plasmid in Shigella flexneri by Cytosine Base Editing
Source: mSystems. 2022 Dec 21;8(1):e01045-22. doi: 10.1128/msystems.01045-22 (PMC9948704; doi:10.1128/msystems.01045-22)
Supplement: TABLE S1 [file msystems.01045-22-st001.docx]

Supplementary Table S1. Bacterial strains, cells and plasmids used in this study.

| Name | Description | Reference |
| --- | --- | --- |
| Bacteria |  |  |
| *E. coli* DH5α | General cloning host |  |
| *E. coli* DB3.1 | *ccdB* resistant strain, cloning host for generating pgRNA_AT |  |
| *E. coli* MG1655 | WT *E. coli* strain |  |
| *E. coli*::*mCherry* | *E. coli* MG1655 *attTn7::mCherry*; *E. coli* MG1655 expressing *mCherry* from the chromosome after integration of *mCherry* at the attTn7 site | This study |
| *S. flexneri* M90T 5a | WT *Shigella flexneri* strain | (1) |
| *S. flexneri*::*mCherry* | *S. flexneri attTn7::mCherry*; *S. flexneri* expressing *mCherry* from the chromosome after integration of *mCherry* at the *attTn7* site | (2) |
| *S. flexneri::mScarlet* | *S. flexneri attTn7::mScarlet*; *S. flexneri* expressing *mScarlet-i* from the chromosome after integration of *mScarlet-i* at the *attTn7* site | This study |
| *S. flexneri* *mxiD(Q323X)* | *S. flexneri* *mxiD* loss-of-function mutant generated by substituting Gln_323_ by STOP codon (C_964_🡪T) | This study |
| *S. flexneri* *icsA(Q59X)* | *S. flexneri* *icsA* loss-of-function mutant generated by substituting Gln_59_ by STOP codon (C_175_🡪T) | This study |
| *S. flexneri* *icsB(Q32X)* | *S. flexneri* *icsB* loss-of-function mutant generated by substituting Gln_32_ by STOP codon (C_94_🡪T) | This study |
| *S. flexneri* *icsB(Q237X)* | *S. flexneri* *icsB* loss-of-function mutant generated by substituting Gln_237_ by STOP codon (C_709_🡪T) | This study |
| *S. flexneri ipgB(Q44X)* | *S. flexneri* *ipgB* loss-of-function mutant generated by substituting Gln_44_ by STOP codon (C_130_🡪T) | This study |
| *S. flexneri* *ipgB(Q190X)* | *S. flexneri* *ipgB* loss-of-function mutant generated by substituting Gln_190_ by STOP codon (C_568_🡪T) | This study |
| *S. flexneri* *vacJ(Q90X)* | *S. flexneri* *vacJ* loss-of-function mutant generated by substituting Gln_90_ by STOP codon (C_268_🡪T) | This study |
| Cells |  |  |
| TC7 cells | Intestinal epithelial cells | (3) |
| Plasmids |  |  |
| pgRNA_ccdB | sgRNA expression plasmid used in *C. glutamicum* | (4) |
| pgRNA_AT | sgRNA expression plasmid for cloning spacers | This study |
| pBluescript SK | General cloning vector used as a source of backbone for pgRNA_AT |  |
| pnCas9-AID-YU | nCas9-AID expressing plasmid used in *C. glutamicum* | (4) |
| pnCas9-AID | nCas9-AID expressing plasmid; carries *sacB* that aids in curing | This study |
| pSU19 | General cloning vector used as a source of backbone for pnCas9-AID | (5) |
| pAS_004 | Intermediate nCas9-AID plasmid before cloning of *sacB* expression cassette | This study |
| pYC1000-eforRED | Source of *sacB* expression cassette | (6) |
| pQE-60NA-mScarlet-2I | Source of *mScarlet-i* | (7) |
| pSU2.1rp-mCherry | Source of *mCherry* expression cassette | (8) |
| pSU2.1rp-cerulean | Plasmid used for cloning *mScarlet-i* (to have the promoter and RBS similar to *mCherry* expression cassette) | (8) |
| pGRG25 | Plasmid for Tn7 transposition | (9) |
| pGRG-mCherry | pGRG25 plasmid with *mCherry* expression cassette cloned for transposition | (2) |
| pGRG-mScarlet | pGRG25 plasmid with *mScarlet-i* expression cassette cloned for transposition | This study |
| pgRNA-(R)-GFP | sgRNA expression plasmid modified to express sfGFP with an RBS | This study |
| pgRNA-X | sgRNA expression plasmid carrying non-targeting spacer | This study |
| pgRNA-m2 | sgRNA expression plasmid carrying mCh2 spacer | This study |
| pgRNA-m3 | sgRNA expression plasmid carrying mCh3 spacer | This study |
| pgRNA-m4 | sgRNA expression plasmid carrying mCh4 spacer | This study |
| pgRNA-icsA_G1 | sgRNA expression plasmid carrying icsA_G1 spacer | This study |
| pgRNA-icsA_G2 | sgRNA expression plasmid carrying icsA_G2 spacer | This study |
| pgRNA_icsA_G3 | sgRNA expression plasmid carrying icsA_G3 spacer | This study |
| pgRNA-icsB_G1 | sgRNA expression plasmid carrying icsB_G1 spacer | This study |
| pgRNA-icsB_G2 | sgRNA expression plasmid carrying icsB_G2 spacer | This study |
| pgRNA-mxiD_G1 | sgRNA expression plasmid carrying mxiD_G1 spacer | This study |
| pgRNA-vacJ_G1 | sgRNA expression plasmid carrying vacJ_G1 spacer | This study |
| pgRNA-vacJ_G2 | sgRNA expression plasmid carrying vacJ_G2 spacer | This study |
| pgRNA-vacJ_G3 | sgRNA expression plasmid carrying vacJ_G3 spacer | This study |

References

1. Sansonetti PJ, Kopecko DJ, Formal SB. 1982. Involvement of a plasmid in the invasive ability of Shigella flexneri. Infect Immun 35:852–860.

2. Tadala L, Langenbach D, Dannborg M, Cervantes-Rivera R, Sharma A, Vieth K, Rieckmann LM, Wanders A, Cisneros DA, Puhar A. 2022. Infection-induced membrane ruffling initiates danger and immune signaling via the mechanosensor PIEZO1. Cell Reports 40:111173.

3. Chantret I, Rodolosse A, Barbat A, Dussaulx E, Brot-Laroche E, Zweibaum A, Rousset M. 1994. Differential expression of sucrase-isomaltase in clones isolated from early and late passages of the cell line Caco-2: evidence for glucose-dependent negative regulation. J Cell Sci 107 ( Pt 1):213–225.

4. Wang Y, Liu Y, Liu J, Guo Y, Fan L, Ni X, Zheng X, Wang M, Zheng P, Sun J, Ma Y. 2018. MACBETH: Multiplex automated Corynebacterium glutamicum base editing method. Metabolic Engineering 47:200–210.

5. Bartolomé B, Jubete Y, Martínez E, de la Cruz F. 1991. Construction and properties of a family of pACYC184-derived cloning vectors compatible with pBR322 and its derivatives. Gene 102:75–78.

6. Yan M-Y, Yan H-Q, Ren G-X, Zhao J-P, Guo X-P, Sun Y-C. 2017. CRISPR-Cas12a-Assisted Recombineering in Bacteria. Appl Environ Microbiol 83:e00947-17.

7. Valbuena FM, Fitzgerald I, Strack RL, Andruska N, Smith L, Glick BS. 2020. A photostable monomeric superfolder green fluorescent protein. Traffic 21:534–544.

8. Campbell-Valois F-X, Sachse M, Sansonetti PJ, Parsot C. 2015. Escape of Actively Secreting Shigella flexneri from ATG8/LC3-Positive Vacuoles Formed during Cell-To-Cell Spread Is Facilitated by IcsB and VirA. mBio 6:e02567-02514.

9. McKenzie GJ, Craig NL. 2006. Fast, easy and efficient: site-specific insertion of transgenes into Enterobacterial chromosomes using Tn7 without need for selection of the insertion event. BMC Microbiol 6:39.
