# Supplementary material for: CRISPR-Cas-Guided Mutagenesis of Chromosome and Virulence Plasmid in Shigella flexneri by Cytosine Base Editing
Source: mSystems. 2022 Dec 21;8(1):e01045-22. doi: 10.1128/msystems.01045-22 (PMC9948704; doi:10.1128/msystems.01045-22)
Supplement: TEXT S1 [file msystems.01045-22-s0001.docx]

### Supplementary Methods S1

#### Construction of fluorescent strains

**F**luorescent strains of *E. coli* and *S. flexneri*, constitutively expressing *mCherry* and *mScarlet-i* (only *S. flexneri*) from the chromosome, were constructed using Tn7-mediated transposition (1, 2). The plasmid pGRG25 expresses Tn7 transposase which stably inserts a single copy of genes cloned between the repeats of the transposon. The insertion event, downstream the *glmS* gene, has no impact on *S. flexneri* virulence (assessed by gentamicin protection assay, data not shown). *mScarlet-i* was amplified from pQE-60NA-mScarlet-2I using the oligos AT_0157 and AT_0158. The PCR product was digested with BamHI and HindIII and ligated to similarly digested pSU2.1rp-cerulean plasmid, yielding pSU-mScarlet plasmid. *mCherry* and *mScarlet-i* expression cassette (the ORF along with a constitutive promoter and ribosome binding site) was amplified from the plasmid pSU2.1rp-mCherry (3), and pSU-mScarlet, respectively, using the primers AT_0017 and AT_0018. The purified PCR product was digested with NotI and XhoI restriction enzymes and ligated to similarly digested pGRG25 plasmid. The resulting plasmids, pGRG-mCherry or pGRG-mScarlet, were transformed into *E. coli* MG1655 and *S. flexneri* M90T 5a, respectively. The transformants were selected on LA (for *E. coli*) or TSA (for *S. flexneri*) plates supplemented with carbenicillin (100 μg/ml) at 30°C. A single transformant colony was grown overnight in LB or TSB supplemented with carbenicillin at 30°C (since pGRG plasmids have a temperature sensitive pSC101 origin). The overnight cultures were diluted in fresh medium containing carbenicillin and the bacteria were grown till OD_600_ = 0.2 at 30°C. Transposition was induced by the addition of 0.5% L-arabinose and the cultures were grown overnight at 30°C. Dilutions of these cultures were spread on agar plates with no antibiotics and incubated overnight at 37°C, since pGRG plasmids cannot replicate at this temperature. The resulting colonies were checked for fluorescence and the fluorescent colonies were patched onto agar plates containing carbenicillin to ensure the loss of pGRG-mCherry (or pGRG-mScarlet). *S. flexneri* colonies were also patched onto Congo red plates (TSA supplemented with 0.01% Congo red) to select only for the virulent colonies. The chromosomal integration of *mCherry* and *mScarlet-i* was verified by sequencing the PCR product obtained by using primers AT_0059 and AT_0061 for *E. coli*, and primers AT_0062 and AT_0063 for *S. flexneri*.

#### Plasmid construction

**T**he sgRNA expression plasmid (pgRNA_AT) was generated by combining parts from pgRNA-ccdB and a pBluescript SK variant that lacks BsaI site in the β-lactamase gene (4). Using the primers AT_0001 and AT_0002, *ccdB* gene and the downstream guide RNA scaffold were amplified from pgRNA_ccdB. The AT_0001 has a synthetic constitutive promoter, J23119, at the 5’ end, making the resulting PCR product a complete sgRNA expression cassette. Using the primers AT_0004 and AT_0005, the plasmid backbone (origin and resistance marker) was amplified from pBluescript SK. Both these PCR products were digested with EcoRI and HindIII and ligated. The resulting chimeric plasmid was amplified using the primers AT_0087 and AT_088 and the PCR product was phosphorylated and self-ligated to form the pgRNA_AT plasmid. The sgRNA cassette in pgRNA_AT, as a result, has an extra C residue downstream the BsaI restriction site to ensure accurate cloning of spacer oligos. *E. coli* DB3.1 was used as a cloning host for generating pgRNA_AT as it is resistant to the action of *ccdB*. To verify the activity of J23119 promoter in *Shigella*, sfGFP was cloned under the promoter in pgRNA_AT. sfGFP encoding gene was amplified from the genomic DNA of sfGFP expressing *Bacteroides thetaiotaomicron* (5) using the primers AT_0057 and AT_0058. The PCR product was digested with BamHI and HindIII and ligated to similarly digested pgRNA_AT to yield pgRNA-(R)-GFP (Supplementary Fig. S1). The plasmid was transformed into WT *S. flexneri* and the transformants were selected on TSA plates supplemented with carbenicillin. A single transformant colony of *S. flexneri* carrying pgRNA-X and pgRNA-(R)-GFP was streaked on a TSA plate containing carbenicillin. The plates were incubated overnight at 37°C and imaged using ChemiDoc (Bio-Rad).

**T**he nCas9-AID expressing plasmid, pnCas9-AID, was generated by combining parts of pSU19 and pnCas9-AID-YU by *in-vivo* assembly (IVA) cloning (4, 6). From pnCas9-AID_YU, the nCas9-AID encoding gene, with *tac* promoter and the LacI encoding gene were amplified using the primers AT_0043 and AT_0044. The p15A origin and antibiotic resistance marker were amplified from pSU19 using the primers AT_0008 and AT_0009. Both PCR products were treated with DpnI (to digest the parent plasmids) and an equimolar mixture of both PCR products was transformed into ultracompetent *E. coli* DH5α. The resulting plasmid, named pAS_004, was sequenced to verify the construction. A *sacB* expression cassette was amplified from pYC1000-eforRED plasmid and cloned in the NotI site of pAS_004. The resulting plasmid was designated as pnCas9-AID.

#### Guide RNA design and cloning

**T**he guide RNA for mutagenesis were designed using the program CRISPR_CBEI which detects the mutable sites and provides the sequences of spacer oligos (7). The editing window was set from -16 to -20 from an ‘NGG’ PAM. We gave preference to mutable sites in the first 50% of the target gene sequence to maximize the odds of creating loss-of-function mutations. The location of the target sequence within the gene can be easily determined from the output of the CRISPR_CBEI program. Multiple mutable sites were found for *mCherry*, *icsA*, *icsB*, and *vacJ* in the first half of the sequence. In case of *ipgB*, the second mutable site was very close to the C-terminus. We still included in the experiments to see whether the phenotype of both mutants was similar. In case of *mxiD*, however, only one mutable site was found, which was located in the second half of the sequence (but resulted in loss-of-function mutants). For every spacer, two complementary oligos were ordered with the forward oligo carrying 5’ – CTAGT and the reverse oligo carrying 5’ – AAAC and a 3’ – A residues. The forward and reverse oligos were annealed in an annealing buffer (10 mM Tris-HCl pH 7.5, 50 mM NaCl, and 1 mM EDTA) by incubating at 95°C for 3 min and letting the mixture cool down to room temperature gradually. The double stranded spacer oligos were 5’ phosphorylated using T4 DNA T4 Polynucleotide Kinase (Thermo Fisher Scientific) and ligated to BsaI digested pgRNA_AT using T4 DNA Ligase (Thermo Fisher Scientific). The ligation product was transformed into *E. coli* DH5α, to obtain target-specific sgRNA plasmids. These plasmids were verified by DNA sequencing and named according to the sgRNA they express (e.g. pgRNA_m2 expresses the sgRNA_m2).

#### Statistical analysis

All statistical analyses were performed in GraphPad Prism. The type of analysis and test of significance is mentioned in the respective figure legends. The default P value system of GraphPad Prism was used in all cases and the key is described in the figure legends.

### References (only for Supplementary Methods S1)

1. McKenzie GJ, Craig NL. 2006. Fast, easy and efficient: site-specific insertion of transgenes into Enterobacterial chromosomes using Tn7 without need for selection of the insertion event. BMC Microbiol 6:39.

2. Tadala L, Langenbach D, Dannborg M, Cervantes-Rivera R, Sharma A, Vieth K, Rieckmann LM, Wanders A, Cisneros DA, Puhar A. 2022. Infection-induced membrane ruffling initiates danger and immune signaling via the mechanosensor PIEZO1. Cell Reports 40:111173.

3. Campbell-Valois F-X, Sachse M, Sansonetti PJ, Parsot C. 2015. Escape of Actively Secreting Shigella flexneri from ATG8/LC3-Positive Vacuoles Formed during Cell-To-Cell Spread Is Facilitated by IcsB and VirA. mBio 6:e02567-02514.

4. Wang Y, Liu Y, Liu J, Guo Y, Fan L, Ni X, Zheng X, Wang M, Zheng P, Sun J, Ma Y. 2018. MACBETH: Multiplex automated Corynebacterium glutamicum base editing method. Metabolic Engineering 47:200–210.

5. Whitaker WR, Shepherd ES, Sonnenburg JL. 2017. Tunable Expression Tools Enable Single-Cell Strain Distinction in the Gut Microbiome. Cell 169:538-546.e12.

6. García-Nafría J, Watson JF, Greger IH. 2016. IVA cloning: A single-tube universal cloning system exploiting bacterial In Vivo Assembly. Sci Rep 6:27459.

7. Yu H, Wu Z, Chen X, Ji Q, Tao S. 2020. CRISPR-CBEI: a Designing and Analyzing Tool Kit for Cytosine Base Editor-Mediated Gene Inactivation. mSystems 5:e00350-20.
