# Supplementary material for: CRISPR-Cas-Guided Mutagenesis of Chromosome and Virulence Plasmid in Shigella flexneri by Cytosine Base Editing
Source: mSystems. 2022 Dec 21;8(1):e01045-22. doi: 10.1128/msystems.01045-22 (PMC9948704; doi:10.1128/msystems.01045-22)
Supplement: TABLE S2 [file msystems.01045-22-st002.docx]

Supplementary Table S2. Oligonucleotides used in this study

| **Name** | **Sequence** | **Purpose** |
| --- | --- | --- |
| AT_0001 | ATTAGAATTCTTGACAGCTAGCTCAGTCCTAGGTATAATACTAGTGAGACCACGCGTGGATCC | Amplification of gRNA expression cassette from pgRNA_ccdB (ref), the forward primer introduces J23119 promoter |
| AT_0002 | GATCAAGCTTGGAGGTCGAAGCCGCACG |  |
| AT_0004 | GTACAAGCTTTGAGCAAAAGGCCAGCAAAAG | Amplification of the plasmid backbone from pBlueScript II |
| AT_0005 | ATCGGAATTCAATGTGCGCGGAACCCC |  |
| AT_0008 | GCATCCTAGGTGCTTTTGCCGTTACGCAC | Amplification of the plasmid backbone from pSU19 |
| AT_0009 | ATTAGCGGCCGCTAATTGCGTTGCGCTCACTG |  |
| AT_0017 | TTATGCGGCCGCGCGTTGGCCGATTC | Amplification of *mCherry* cassette from pSU2.1rp-mCherry |
| AT_0018 | ATTACTCGAGCCAGGGTTTTCCCAGTC |  |
| AT_0043 | GCGCAACGCAATTAGCGGCCGCTAATGCATCCGGGGCTGATCCCCG | Amplification of dCas9-AID from pCas9-AID (ref) for IVA cloning |
| AT_0044 | GCGTAACGGCAAAAGCACCTAGGATGCGGCCATCCGTCAGGATGGCC |  |
| AT_0057 | ATTAGGATCCGAAAGAGGAGAAATACTAGATGGATTCAAAAAAATTTAAAATAATGCG | Cloning *gfp* (with RBS) in pgRNA |
| AT_0058 | TAGCAAGCTTAACCTTTATCATCATCGTCC |  |
| AT_0059 | TTCCTGGGCCGTGGCGATC | Sequencing and verification of *E. coli*::*mCherry* |
| AT_0061 | TACCCTGGTAGTTAACTTTATTACCGG |  |
| AT_0062 | CCGTTCCGCTGCAGCTG | Sequencing and verification of *S. flexneri*::*mCherry* |
| AT_0063 | ACACAGCCACTGGATCGG |  |
| AT_0087 | CCGTTTTAGAGCTAGAAATAGCAAG | Modification of the pgRNA cloning site by adding a C residue |
| AT_0088 | AGACCTTATATTCCCCAGAACATCAG |  |
| AT_0093 | CTAGTTCAGTTCATGTACGGCTCCA | Cloning mCh2 spacer in pgRNA |
| AT_0094 | AAACGTGGAGCCGTACATGAACTGA |  |
| AT_0095 | CTAGTGACCCAGGACTCCTCCCTGC | Cloning mCh3 spacer in pgRNA |
| AT_0096 | AAACGCAGGGAGGAGTCCTGGGTCA |  |
| AT_0097 | CTAGTGCTCCCACTTGAAGCCCTCG | Cloning mCh4 spacer in pgRNA |
| AT_0098 | AAACGCGAGGGCTTCAAGTGGGAGC |  |
| AT_0099 | CTAGTGGATCCACTAGTGCGGCCGC | Cloning gRNA-X in pgRNA |
| AT_0100 | AAACGGCGGCCGCACTAGTGGATCC |  |
| AT_0105 | CTAGTCATAAACCGCCGAGTCACCA | Cloning gyrA spacers in pgRNA |
| AT_0106 | AAACTGGTGACTCGGCGGTTTATGA |  |
| AT_0109 | CTAGTTGGCAAGGAACGGCTTCATT | Cloning mxiD_G1 spacers in pgRNA |
| AT_0110 | AAACAATGAAGCCGTTCCTTGCCAA |  |
| AT_0111 | CTAGTGACCCAATGTTCACAGGGGG | Cloning icsA_G1 spacers in pgRNA |
| AT_0112 | AAACCCCCCTGTGAACATTGGGTCA |  |
| AT_0113 | CTAGTCAATGTTCACAGGGGGGGGC | Cloning icsA_G2 spacers in pgRNA |
| AT_0114 | AAACGCCCCCCCCTGTGAACATTGA |  |
| AT_0115 | CTAGTTCAAGAACTTCATTTTTCAG | Cloning icsA_G3 spacers in pgRNA |
| AT_0116 | AAACCTGAAAAATGAAGTTCTTGAA |  |
| AT_0119 | CTAGTCTTGCAGGGCGACCCTTATC | Cloning vacJ_G1 spacers in pgRNA |
| AT_0120 | AAACGATAAGGGTCGCCCTGCAAGA |  |
| AT_0121 | CTAGTTTGCAGGGCGACCCTTATCA | Cloning vacJ_G2 spacers in pgRNA |
| AT_0122 | AAACTGATAAGGGTCGCCCTGCAAA |  |
| AT_0123 | CTAGTTGCAGGGCGACCCTTATCAG | Cloning vacJ_G3 spacers in pgRNA |
| AT_0124 | AAACCTGATAAGGGTCGCCCTGCAA |  |
| AT_0137 | CTAGTAGCACAGAAATTCAACCTAA | Cloning icsB_G1 spacers in pgRNA |
| AT_0138 | AAACTTAGGTTGAATTTCTGTGCTA |  |
| AT_0141 | CTAGTAATCAAAAAAAAGACCCCTA | Cloning icsB_G2 spacers in pgRNA |
| AT_0142 | AAACTAGGGGTCTTTTTTTTGATTA |  |
| AT_0143 | ACATATGCTCACGAGGTACA | Sequencing *icsB* |
| AT_0144 | CCATACCAGCACAGTTTTC |  |
| AT_0147 | ATTGCATATCCAGAAACC | Sequencing *mxiD* |
| AT_0148 | TAGCCGGAATATTCTCTTG |  |
| AT_0151 | GGAACTACGCTTCTGGTG | Sequencing *vacJ* |
| AT_0152 | CGTGAAGCTACCGTAGAAC |  |
| AT_0153 | AGGTAAATTTCTCCCGTTG | Sequencing *icsA* |
| AT_0154 | TGATTACAGAGAGGCTGCT |  |
| gRNA-X | GGATCCACTAGTGCGGCCGC | Random non-binding spacer |
| mCh2 | TCAGTTCATGTACGGCTCCA | *mCherry* spacer for C_205_🡪T, Gln_69_🡪STOP mutation |
| mCh3 | GACCCAGGACTCCTCCCTGC | *mCherry* spacer for C_340_🡪T, Gln_114_🡪STOP mutation |
| mCh4 | GCTCCCACTTGAAGCCCTCG | *mCherry* spacer for G_294_🡪A, Trp_98_🡪STOP mutation |
| icsA_G1 | GACCCAATGTTCACAGGGGG | *icsA* spacer for C_37_🡪T, Gln_13_🡪STOP mutation |
| icsA_G2 | CAATGTTCACAGGGGGGGGC | *icsA* spacer for C_175_🡪T, Gln_59_🡪STOP mutation |
| icsA_G3 | TCAAGAACTTCATTTTTCAG | *icsA* spacer for C_442_🡪T, Gln_148_🡪STOP mutation |
| icsB_G1 | AGCACAGAAATTCAACCTAA | *icsB* spacer for C_94_🡪T, Gln_32_🡪STOP mutation |
| icsB_G2 | AATCAAAAAAAAGACCCCTA | *icsB* spacer for C_709_🡪T, Gln_237_🡪STOP mutation |
| mxiD_G1 | TGGCAAGGAACGGCTTCATT | *mxiD* spacer for C_964_🡪T, Gln_323_🡪STOP mutation |
| vacJ_G1 | CTTGCAGGGCGACCCTTATC | *vacJ* spacer for C_268_🡪T, Gln_90_🡪STOP mutation |
| vacJ_G2 | TTGCAGGGCGACCCTTATCA | *vacJ* spacer for C_268_🡪T, Gln_90_🡪STOP mutation |
| vacJ_G3 | TGCAGGGCGACCCTTATCAG | *vacJ* spacer for C_268_🡪T, Gln_90_🡪STOP mutation |
| sgRNA-gyrA | CATAAACCGCCGAGTCACCA | *gyrA* spacer for G_259_🡪A, Asp_87_🡪Asn mutation |
